# Effect of *Porcine circovirus 2* (PCV-2) maternally derived antibodies on performance and PCV-2 viremia in vaccinated piglets under field conditions

**DOI:** 10.1186/s40813-019-0128-7

**Published:** 2019-09-05

**Authors:** S. Figueras-Gourgues, L. Fraile, J. Segalés, I. Hernández-Caravaca, R. López-Úbeda, F. A. García-Vázquez, O. Gomez-Duran, B. Grosse-Liesner

**Affiliations:** 1 Department of Physiology, Faculty of Veterinary, Campus Mare Nostrum, University of Murcia, 30100 Murcia, Spain; 2 IMIB-Arrixaca, Murcia, Spain; 3 Departamento de Ciencia Animal, Universidad de Lleida, Lleida, Spain; 4 Departament de Sanitat i Anatomia Animals, Facultat de Veterinària, Universitat Autònoma Barcelona, 08193 Bellaterra, Spain; 5 UAB, Centre de Recerca en Sanitat Animal (CRESA, IRTA-UAB), Campus de la Universitat Autònoma de Barcelona, 08193 Bellaterra, Spain; 6 Department of Cell Biology and Histology, Faculty of Medicine, Campus Mare Nostrum, University of Murcia, 30100 Murcia, Spain; 7 Boehringer Ingelheim Vetmedica GmbH AH Swine, Ingelheim, Germany

**Keywords:** Porcine circovirus type 2, Piglets, Vaccine

## Abstract

**Background:**

Nowadays, the most common presentation of PCV-2 is the subclinical infection in piglets after weaning. The success of PCV-2 vaccination is associated with the control of the clinical disease as well as the improvement of production parameters. In consequence, the objective of the present study was to analyse the effect of PCV-2 maternally derived antibody (MDA) levels on vaccine efficacy in piglets vaccinated at three weeks of age with a commercial PCV-2 subunit vaccine. The study was performed analysing a database with 6112 wean-to-slaughter piglets from 4 different European regions.

**Results:**

Results showed that the use of the vaccine was able to decrease the PCV-2 viremia calculated as area under the curve (AUC = 60.29 ± 3.73), increase average daily weight gain (ADWG = 0.65 ± 0.01 kg/day) and reduce mortality (7%) in vaccinated piglets compared to non-vaccinated ones (AUC of 198.27 ± 6.14, 0.62 ± 0.01 kg/day and 11% respectively). The overall difference of ADWG between both groups was close to 30 g per day (*p* < 0.05), also when they were split for low and high levels of MDA titres. Moreover, the animals with the highest ADWG were observed in the group of piglets vaccinated with high or extremely high antibody titres (0.66 and 0.65 kg/day respectively). Considering only animals with extremely high antibody titres, both study groups performed similar, however there was a numerical difference of 10 g/day in favour of vaccinated piglets. Likewise, lack of correlation between ADWG and MDA was observed suggesting that no maternal antibody interference was present with the tested vaccine because the vaccinated animals grew faster compared to unvaccinated control animals, regardless of the level of maternal antibodies present at the time of vaccination.

**Conclusions:**

The results of the present study demonstrated that the MDA against PCV-2 transferred through the colostrum intake has a protective effect against this viral infection. The vaccine used in the present study (Ingelvac CircoFLEX®) was effective when applied at three weeks of age and was not affected by the level of MDA at the time of vaccination.

## Background

Porcine circovirus type 2 (PCV-2) has been associated with a number of clinical conditions in swine that can cause high economic losses for the pig industry [1, 2]. The most common clinical manifestation of these diseases has been post-weaning multisystemic wasting syndrome (PMWS), which was first described in Canada in 1991 [3, 4]. The term porcine circovirus diseases (PCVDs) has been used to compile all conditions linked to PCV-2 [5], and subsequently these clinical syndromes were reviewed and some new names proposed [6]. Nowadays the most common presentation of PCV-2 is the subclinical infection (PCV-2-SI) in piglets after weaning. Other described syndromes are: PCV-2 systemic disease (PCV-2-SD, the former PMWS) [7–9], PCV-2 lung disease (PCV-2-LD), PCV-2 enteric disease (PCV-2-ED) [10–13], PCV-2 reproductive disease (PCV-2-RD) [14–16] and porcine dermatitis and nephropathy syndrome (PDNS) [17]. However, PCV-2-LD and PCV-2-ED have been considered as negligible conditions, since the virus linked with lung and intestine inflammation is usually found within the framework of PCV-2-SD [18, 19].

The success of PCV-2 vaccination is associated with the control of the clinical disease as well as prevention of tissue lesions [1, 20–23]. Even when no overt clinical signs are observed in PCV-2-SI scenarios, several field studies have demonstrated that PCV-2 piglet vaccination is able to improve production parameters [average daily weight gain (ADWG)], percentage of runts, body condition and carcass weight) [24]. In consequence, the use of vaccination in farms with clinical PCVD and PCV-2-SI has shifted the general pig health from a period of worldwide severe clinical outbreaks (1997–2007) to self-limiting subclinical infections with occasional outbreaks. In this widespread vaccination scenario in piglets and the increasing use of PCV-2 vaccines in sows, the maternally derived antibody (MDA) levels and their potential interference with vaccines applied in their offspring [25] might pose a risk to vaccination efficacy. Therefore, it may be necessary to refine the vaccination timing schedules commonly established around weaning for piglets.

Different laboratory tests may be used for the detection of PCV-2 antibodies in serum. Therefore, serology can be regarded as a useful tool for monitoring presence of antibodies in pigs and sows, and potentially assess their levels at the time of vaccination. Traditionally, the IPMA (immunoperoxidase monolayer assay) method has been used to detect PCV-2 antibodies quantitatively. However, Guedes et al. [26] showed that the IFAT (indirect fluorescence antibody test) method for the diagnosis of the porcine proliferative enteropathy was able to offer very similar values to the IPMA (98,6% of agreement). PCV-2 antibody levels have been described to be moderate to high in piglets when above 1:320 or its equivalent 2.5 log_10_ using indirect fluorescence antibody titration (IFAT) [27].

The objective of the present study was to analyse the effect of PCV-2 MDA levels on vaccine efficacy (measured by ADWG and PCV-2 viremia) in piglets vaccinated at three weeks of age with a commercial PCV-2 subunit vaccine. The study was performed in four different farms in Europe.

## Results

### Descriptive analysis of the effect of vaccination

**Fig. 1:**
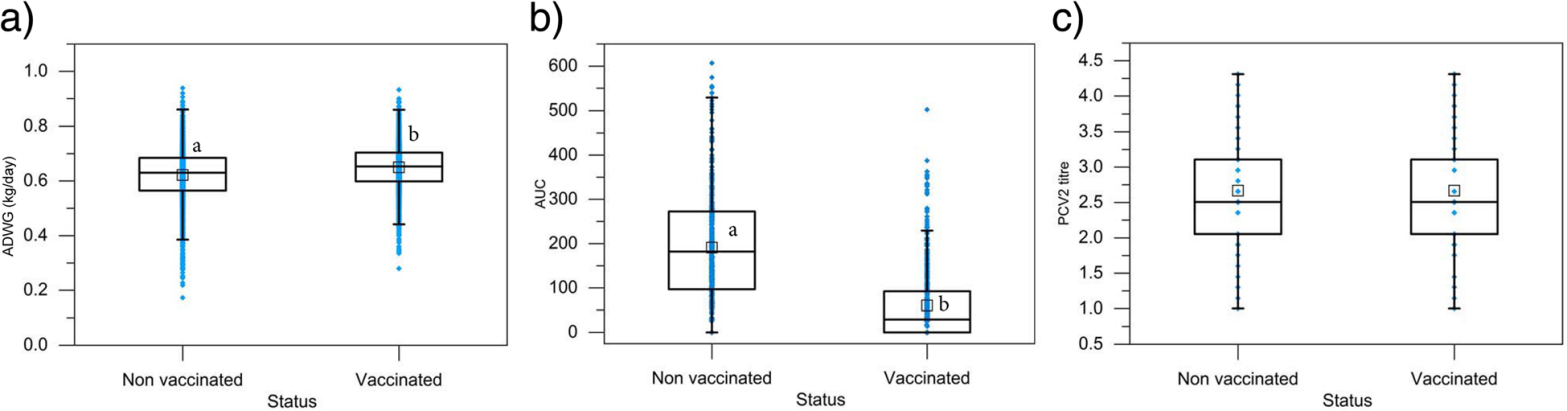
Effect of vaccination on average daily weight gain (ADWG), PCV-2 viral load (represented as area under curve-AUC) and PCV-2 antibody titre. **a**) Comparison of the average daily weight gain (ADWG, kg/day) in placebo-treatment (*n* = 2728) and vaccinated animals (*n* = 2835), **b**) comparison of the PCV-2 viral load as area under curve (AUC) in placebo-treatment (*n* = 484) and vaccinated animals (*n* = 472) and **c**) comparison of the PCV-2 antibody titre (log_10_) in placebo-treatment (*n* = 2728) and vaccinated animals (*n* = 2835). Different letters (**a**, **b**) indicate significant differences (*P* < 0.05)

The first objective was to analyse the effect of the vaccine on each of the parameters studied independently (Fig. 1). The mean ADWG was significantly higher in the vaccinated animals (0.65 ± 0.01 kg/day) compared with the placebo-treated animals (0.62 ± 0.01 kg/day), corresponding to an overall ADWG difference between the groups of 30 g per day (Fig. 1a; *p* < 0.05). The same result was observed in all farms used in this study; in all of them ADWG was significantly higher in the vaccinated animals (Table 1; p < 0.05), with a range of ADWG differences between groups between 20 and 50 g per day.

**Table 1:** Mean of ADWG (kg/day) ± SEM for each of the four studies analysed

Treatment	Southern Germany [1]	Northern Germany [28]	United Kingdom [29]	France [30]
Placebo animals (*n* = 2728)	0.61 ± 0.01 ^a^ (*n* = 654)	0.65 ± 0.01 ^a^ (*n* = 706)	0.56 ± 0.01 ^a^ (*n* = 657)	0.66 ± 0.01 ^a^ (*n* = 711)
Vaccinated animals (*n* = 2835)	0.64 ± 0.01 ^b^ (*n* = 677)	0.67 ± 0.01 ^b^ (*n* = 718)	0.61 ± 0.01 ^b^ (*n* = 718)	0.68 ± 0.01 ^b^ (*n* = 722)
ADWG difference between groups (g/day)	30	20	50	20

n: number of animals. ^a,b^ Different letters in the same column indicate significant differences (*p* < 0.05). Only the surviving animals per treatment group were used

The mean value of area under the curve (AUC) in vaccinated animals (60.29 ± 3.73) was significantly (p < 0.05) lower than that of unvaccinated animals (198.27 ± 6.14). The profile of PCV-2 viral load as AUC at the end of the study is illustrated in Fig. 1b. The overall AUC in vaccinated animals was 3.3 times lower than that of placebo-treated animals (p < 0.05).

MDA titres were determined from all animals the day of vaccination. Prior to vaccination PCV-2 antibody titres of both treatment groups were statistically equal in both study groups with a mean geometric value of 2.7 log_10_ (Fig. 1c; *p* > 0.05). The same result was observed in all farms used in this study; in all of them MDA was significantly similar in the vaccinated animals vs. placebo-treated animals (Table 2; p > 0.05).

**Table 2:** Mean of MDA (PCV-2 antibody titre (log_10_)) ± SEM for each of the four studies analysed

Treatment	Southern Germany [1]	Northern Germany [28]	United Kingdom [29]	France [30]
Placebo animals (*n* = 2728)	2.80 ± 0.03 (*n* = 654)	2.87 ± 0.03 (*n* = 706)	2.42 ± 0.02 (*n* = 657)	2.56 ± 0.02 (*n* = 711)
Vaccinated animals (*n* = 2835)	2.83 ± 0.03 (*n* = 677)	2.86 ± 0.03 (*n* = 718)	2.39 ± 0.02 (*n* = 718)	2.60 ± 0.02 (*n* = 722)

n: number of animals. No significant differences found (*p* > 0.05). Only the surviving animals per treatment group were used

### Average daily weight gain (ADWG) according to MDA

**Fig. 2:**
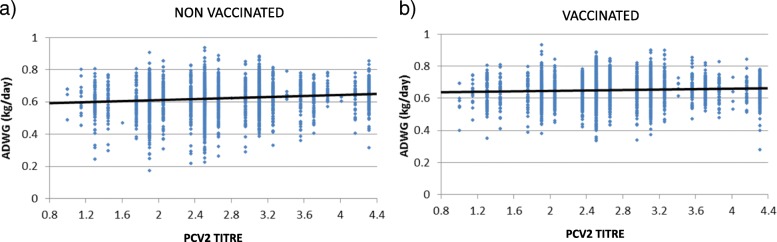
Bivariate adjustment between the average daily weight gain (ADWG) and the PCV-2 antibody titre. Graphs show dates for placebo-treatment (*n* = 2728) **a**) and vaccinated (*n* = 2835) animals **b**). The ADWG of each animal was calculated as the difference in body weight between two measurement periods (4 and 25 weeks of age) divided by the number of days between them

This analysis was done in order to investigate whether the presence of MDA against PCV-2 had any influence on the efficacy of vaccination, in terms of average daily weight gain (ADWG). Figure 2 shows the ADWG depending on the MDA titres in vaccinated and placebo-treated animals. No significant correlation (*p* > 0.05) was observed between these two variables for both treatment groups.

**Fig. 3:**
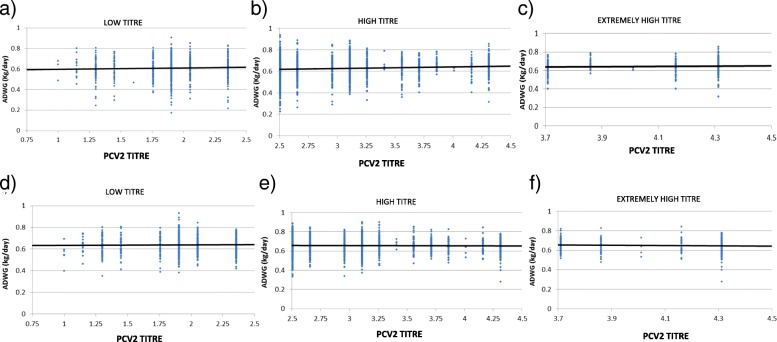
Bivariate adjustment between average daily weight gain (ADWG) and PCV-2 antibody titre divided by PCV-2 titres. Graphs show dates for placebo-treatment (n = 2728) and vaccinated animals (n = 2835). Analysis divided by PCV-2 titres **a**) placebo low titre (< 2.5 log_10_; *n* = 811), **b**) placebo high titre (≥2.5 log_10_; *n* = 1917), **c**) placebo extremely high titre (≥3.7 log_10_; *n* = 300), **d**) vaccinated low titre (< 2.5 log_10_; *n* = 877), **e**) vaccinated high titre (≥2.5 log_10_; *n* = 1958) and **f**) vaccinated extremely high titre (≥3.7 log_10_; *n* = 334)

The levels of MDA recorded for all animals on the day of inclusion (3 weeks of age), were used to evaluate the distribution of antibody levels in the study population. The animals were split in low (< 2.5 log_10_), high (≥2.5 log_10_) and extremely high (≥3.7 log_10_) PCV-2 titres at the time of vaccination. Figure 3 shows a lack of correlation between ADWG and MDA of vaccinated or non-vaccinated animals according to this grouping (*p* > 0.05).

When the ADWG was analysed comparing vaccinated and non-vaccinated animals for low (< 2.5 log_10_) and high (≥2.5 log_10_) PCV-2 antibody titres at vaccination (Table 3), ADWG was significantly higher in the vaccinated animals vs the control ones for both groups (low: 0.64 vs. 0.61 kg/day, respectively; high: 0.66 vs. 0.62 kg/day, respectively; *p* < 0.05). In contrast, no significant differences (p > 0.05) were found between the experimental groups (vaccinated vs non-vaccinated) after analysing the population of animals with extremely high PCV-2 antibody titres (≥3.7 log_10_). However, a numerical difference (10 g) was observed in favour of vaccinated animals (0.65 vs. 0.64 kg/day, respectively) and it must be taken into account the low number of animals in this group that decreases significantly the statistical potency. It is important to note that for both study groups the lowest levels of ADWG appear in the group of low PCV-2 antibody titres.

**Table 3:** Mean of ADWG (kg/day) for placebo and vaccinated animals according to the levels of PCV-2 antibody titre

PCV2 titre	Placebo animals (*n* = 2728)	Vaccinated animals (*n* = 2835)
Low titre (< 2.5 log_10_)	0.61 ^a,A^ (n = 811)	0.64 ^a,B^ (n = 877)
High titre (≥2.5 log_10_)	0.62 ^b,A^ (*n* = 1617)	0.66 ^b,B^ (*n* = 1624)
Extremely high titre (≥3.7 log_10_)	0.64 ^c,A^ (n = 300)	0.65 ^b,A^ (n = 334)

^a,b,c^ Different letters in the same column indicate significant differences (*p* < 0.05)

^A, B^ Different letters in the same row indicate significant differences (*p* < 0.05). Only the surviving animals per treatment group were used

After analysing the ADWG for the different levels of PCV-2 antibody titres (low, high and extremely high) in each one of the study groups (placebo vs. vaccinated animals), it is observed that in the case of the placebo animals there are statistical differences between all the levels (0.61, 0.62 and 0.64) whereas in the vaccinated animals only statistical differences appear between the low level and the rest, there being no differences between the somewhat high and extremely high levels (0.66 vs. 0.65).

Because of these results, two further analyses were carried out to evaluate in depth the group of extremely high antibody titres. For the first one, those animals with PCV-2 antibody titres ≥4.31 log_10_ (highest antibody titres) were compared for both study groups (placebo-treatment *n* = 158 and vaccinated animals *n* = 163), without finding significant differences between them (*p* > 0.05). For the second analysis, animals with highest antibody titres (≥4.31 log_10_; n = 163) were compared to animals with lowest antibody titres (≤1.90 log_10_; *n* = 202) and only within the vaccinated group finding numerically superior (10 g; p > 0.05) differences for the highest antibody titre group.

### PCV-2 AUC level according to MDA

When de AUC was analysed for the different levels of PCV-2 antibody titres (low, high and extremely high) in each one of the study groups (placebo vs. vaccinated animals) (Table 4), it is observed that in the case of the placebo animals only statistical differences appear between low and extremely high levels (225.0 vs. 147.6) whereas in the vaccinated animals no differences were found between the different levels (63.4, 55.4 and 75.5). After comparing the study groups for each of the antibody levels, it is observed that in all cases the viremia is statistically higher for the placebo animals compared to the vaccinated animals (low: 225.0 vs. 63.4; high: 188.5 vs. 55.4 and extremely high: 147.6 vs. 75.5, respectively; *p* < 0.05).

**Table 4:** Mean of area under the curve (AUC) of PCV2 load over time for placebo and vaccinated animals according to the levels of PCV-2 antibody titre

PCV2 titre	Placebo animals (*n* = 472)	Vaccinated animals (*n* = 484)
Low titre (< 2.5 log_10_)	225.0 a,A (*n* = 169)	63.4 a,B (*n* = 173)
High titre (≥2.5 log_10_)	188.5 ab,A (*n* = 265)	55.4 a,B (*n* = 262)
Extremely high titre (≥3.7 log_10_)	147.6 b,A (*n* = 38)	75.5 a,B (*n* = 49)

^a,b^ Different letters in the same column indicate significant differences (*p* < 0.05)

^A, B^ Different letters in the same row indicate significant differences (p < 0.05). Only 15% of randomly pre-selected study animals, chosen as representative sample animals, were used

### Average daily weight gain (ADWG) according to PCV-2 AUC level

**Fig. 4:**
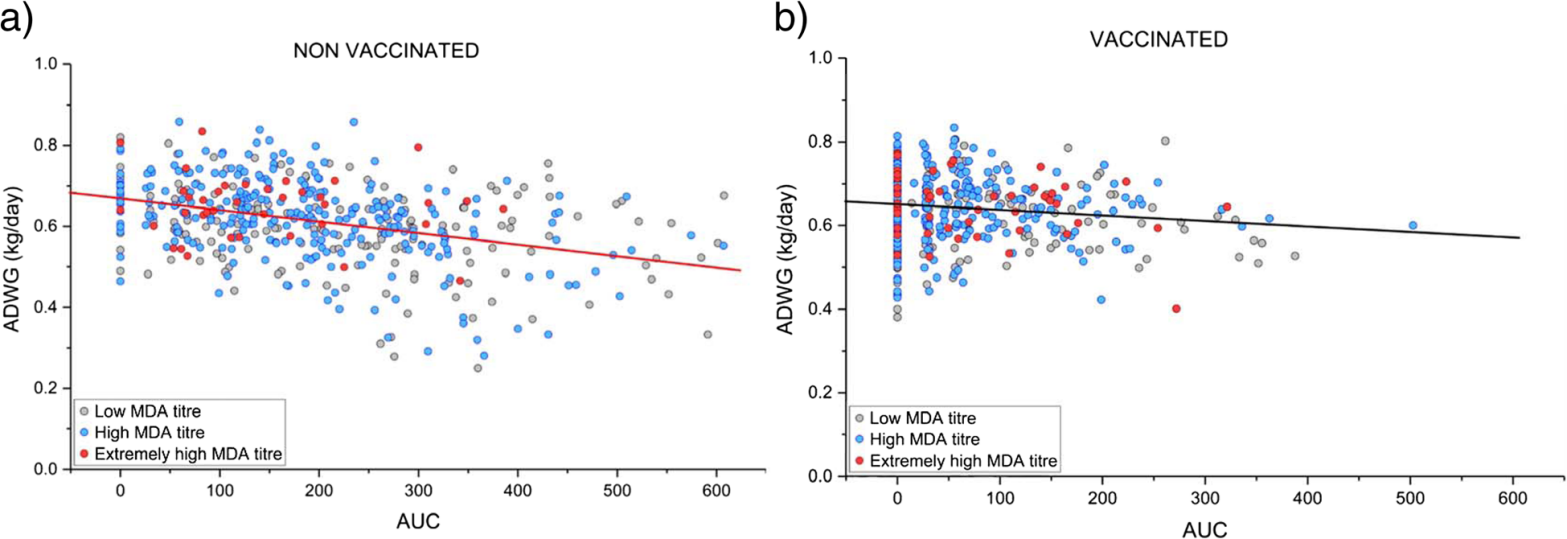
Bivariate adjustment between the average daily weight gain (ADWG) and the viraemia (represented as area under curve-AUC). Graphs show dates for **a**) placebo-treatment (*n* = 472) and **b**) vaccinated animals (*n* = 484)

A significant (p < 0.05) negative correlation (R = − 0.3748) between ADWG and AUC (Fig. 4) was observed for non-vaccinated pigs. Thus, the higher the PCV-2 AUC viremia, the lower the ADWG. This correlation was not observed in vaccinated population (*p* = 0.06).

### Mortality

The mortality within vaccinated animals was always lower than in control animals at farm level, showing statistical significant differences in two of the studies (p < 0.05) (Table 5). Moreover, merging data from the four studies, the mortality in vaccinated animals was significantly lower than in placebo pigs (7% vs. 11%, respectively; p < 0.05) (Table 5). However, it was not observed a significant association between PCV-2 MDA and mortality between farms.

**Table 5:** Percentage of mortality for each of the four studies analysed and for the merged data

Treatment	Southern Germany [1]	Northern Germany [28]	United Kingdom [29]	France [30]	Merged data
Placebo animals (%)	14 ^a^	9	15 ^a^	6	11 ^a^
Vaccinated animals (%)	10 ^b^	7	5 ^b^	5	7 ^b^

^a,b^ Different letters in the same column indicate significant differences (*p* < 0.05). Merged data refers to the grouping of animals of all the farms studied

## Discussion

At the beginning of the 90s, PCV-2-SD emerged as a new swine disease, which was characterized by wasting, a strong reduction of ADWG and high mortality, appearing mainly between 5 and 12 weeks of life [7, 31]. Moreover, the disease caused an increase in the cost of production not only due to mortality and loss of ADWG, but also due to an increase of medication costs and the number of animals with no commercial value [32]. As a consequence, a loss of profitability was observed in affected farms. However, the advent of PCV-2 vaccines by late 2000s demonstrated that the disease is able to be counteracted, and further benefits come from the prevention of subclinical infections [33].

Antibodies of maternal origin are present in all piglets if appropriate colostrum intake takes place [34]; such antibodies in serum gradually decrease during the phases of lactation and nursery [35]. PCV-2 viremia usually appears between the final phase of nursery and the start of the fattening phase, coinciding with the time when MDA reach minimum levels [36, 37], and suggesting therefore that MDA confer some protection against the development of the disease.

Since the very beginning, piglet vaccination was proven to be highly efficacious to control PCV-2-SD as well as PCV-2-SI [28]. However, taking into account that vaccination takes place in presence of maternally derived immunity, the potential interference of MDA with vaccine efficacy has to be considered. Interference with maternal antibodies could be vaccine dependent as the different vaccines available on the market are based on different antigen expression systems and also differ in antigen quantity, purity, and adjuvants. As a working hypothesis, it was expected that the vaccine used in the present study might be less effective in animals with “high” MDA titres. However, the vaccine proved to be effective regardless of the level of maternal antibodies present at the time of vaccination. In consequence, the used vaccine was able to overcome usual levels of MDA, decrease the viremia loads and increase ADWG compared to non-vaccinated piglets [20, 28], with an overall difference of ADWG between groups of 30 g per day both for low and high levels of MDA titres. Moreover, according to obtained results, a maternal antibody interference with the efficacy of the tested vaccine was not observed, since irrespective of the maternal antibody level present at the time of vaccination, the vaccinated animals grew faster compared to the non-vaccinated control animals.

Likewise, the application of the vaccine was able to reduce mortality in vaccinated animals. However, it was not observed a significant association between PCV-2 MDA and mortality between farms, probably because the mortality was due to many factors (infectious and non-infectious ones) and the maternally immunity against PCV2 is only one of them.

The interference of the maternally transferred immunity (MDI) against any vaccine can be described as a blocking of the antigen by the circulating antibodies or by other components, presumably cell mediated, acquired through colostrum. This is well understood for other swine infectious diseases like Aujeszky Disease virus (ADV) [38], Classical Swine Fever virus [39], Influenza A virus (IAV) [40] or Swine Parvovirus [41]. For example, pigs with significant IAV-specific MDA titres can have suppressed adaptive antibody responses to homologous infection or vaccination [40, 42]. For both IAV and ADV evidence suggests that the cellular immune response appears to be less susceptible to MDA [43]. The impact of MDA on active immunisation with PCV2 vaccines in young pigs has been studied previously [44–46]. One of these studies found that under the conditions of that trial that age of vaccination rather than MDA influenced the efficacy of PCVD control. The authors concluded that PCV2 vaccination in the presence of high MDA levels is efficacious when used in 3-week old but not in 1-week old pigs, suggesting that vaccine efficacy was independent of the level of MDA. The authors speculated that other age-related factors may have interfered with the efficacy of the vaccine in 1-week old piglets [44]. Another study using a different PCV2 vaccine found that while interference at the very highest MDA titres could not be excluded the authors concluded that under field conditions this impact is probably negligible [46]. The MDA interference effect could result in a lack of passive immunity but also a lack of active immunisation resulting in worst growing performance of the animals when challenged. In one study with high titres in sows induced by vaccination, it was concluded that some interference of MDA on the induction of an efficient immune response should be considered [45]. They concluded that an optimal PCV2 vaccination strategy needs to balance the levels of passive immunity, the management practices and timing of infection. Contrary to this statement, this study shows that the pigs that had superior ADWG were those that presented high or very high MDA titres at the time of vaccination (low: 0.64; high: 0.66 and extremely: 0.65 kg/day). This observation differs slightly with the results obtained by Feng et al. [46] where the low and high MDA vaccinated piglets (*n* = 78 and 93 respectively) grew equally. This difference could be explained by the size of the groups between both studies. However, the results obtained in the present study could be due to the fact that high titers of antibodies mean high level of protection to the field virus challenge [47]. These animals had an apparent double protection, the one provided by their own high MDA (passive immunity) and the one by vaccination (active immunity which overcomes mostly MDA). It is very likely that, in these circumstances, the antibody levels remain fairly high during the whole life of the animal and, therefore, protected against the natural challenge.

The piglets with a huge amount of antibodies at time of vaccination (≥4.31 log_10_) showed the same or even numerically better growth (10 g; *p* > 0.05) than those animals with the lowest amount of circulating antibodies (≤1.90 log_10_). This fact suggests that the vaccine antigen was not neutralized by MDA even in the highest antibody concentrations, reducing the likelihood of suspected interference. This observation is in line with the results obtained in the Feng et al. [46] study where 10 animals with extremely high MDA were compared to 157 animals with a lower MDA showing no statistical differences in growth.

On the other hand, the present study also shows that in the unvaccinated population the pigs that grew more were those that presented high or very high MDA titres (low: 0.61; high: 0.63 and extremely: 0.64 kg/day) supporting the fact that MDI can provide some degree of protection. Similar results were found in the Feng et al. study [46]. In the case of the groups with extremely high and highest MDA (≥ 3.7 and ≥ 4.31 log_10_ respectively), no statistical differences in ADWG were observed between the vaccinated and non-vaccinated animals. It must be taken into account the low statistical power in these groups selected for extremely high MDA.

These observations reinforce the fact that the MDA transferred through the intake colostrum has a protective effect against PCV-2 infection. Similar results were found in previous studies using the same vaccine used in the present trial [46, 48]. While a vaccine effect in relation to ADWG could not be statistically demonstrated in this sub-population of pigs receiving extremely high MDA, it is also true that no conclusive evidence of interference could be determined. In the case of unvaccinated animals with very and highest MDA titres, they appear to be more protected against PCV-2 and therefore a good growth performance was observed in these groups. The results obtained could be explained by a better early protection in these animals [36] due to the presence of some type of immune factor from colostrum beside antibodies such as lymphocytes, cytokines [34], which could contribute to a better basal immunity. On the other hand, the effect could be confounded by the broad benefit that high intake of colostrum confers to piglets during their whole life [49]. This small sub-population of piglets receiving extremely high MDA might not need an active immunization against PCV2 in the context of a well-protected herd.

However, under a practical vaccination approach, where we cannot determine the MDA status of every pig and cannot assume immunity, the entire piglet population should be vaccinated to ensure homogeneous and full protection. Furthermore, these results may support the vaccination of sows since generating high MDA [50] would protect piglets from early infection and would not interfere with active immunization against PCV-2. The present data supports that vaccination against PCV-2 results in a significant reduction in piglet viraemia irrespective of the initial MDA status, which confirms the positive effect of vaccination. Non-vaccinated animals had higher AUC of viral load and lower ADWG compared to vaccinated ones, which is in accordance with what has been obtained in recent study [48].

## Conclusions

The results of the present study demonstrated that the efficacy (in terms of ADWG improvement and viraemia) of the vaccine used in the present study (Ingelvac CircoFLEX®), when applied at 3 weeks of age to control PCV-2, was not affected by the level of MDA at the time of vaccination.

## Materials and methods

### Farm description and situation

The present study was performed using data from four field trials conducted in different locations of Europe (southern and northern of Germany [1, 28], United Kingdom [29] and France [30]). A total of 6112 conventional commercial (1514 southern and 1542 northern of Germany; 1536 United Kingdom and 1520 France) cross-bred piglets from non-vaccinated sows against PCV-2 were used in this work.

These four studies were conducted 10 years ago when the disease was in the epizootic phase, so the farms got infected approximately at the same time and clinical signs were observed and PCV-2-systemic disease diagnosed according accepted criteria. In three studies [1, 29, 30] a consistent PCV-2 systemic disease (PCV-2 SD) with wasting as a major clinical sign was described. In the other study [28], most of the clinical signs were respiratory, but PCV-2-systemic disease was also diagnosed.

### Test articles

An inactivated subunit vaccine (Ingelvac CircoFLEX®, Boehringer Ingelheim Vetmedica GmbH) was administered at three weeks of age for immunization against PCV-2. The vaccine contained the ORF2 capsid protein of PCV-2 as active component and an aqueous polymer (carbomer) as adjuvant. This protein has been identified as the major immunogenic antigen of PCV-2 inducing a protective response [51, 52]. The ORF2 sequence was subsequently inserted into a baculovirus expression system using an insect cell line derived from ovaries of the armyworm *Sodoptera frugiperda* (SF+ cells) as host. The placebo control consisted of insect cell culture supernatant without PCV-2 capsid protein but containing carbomer adjuvant.

### Study design

All 4 field trials were performed according to the principles of “Good Clinical Practice” (GCP) and followed a randomized, negative-controlled, double-blinded, parallel study design. All piglets enrolled into the field studies received a single dose (1 mL) of the PCV-2 vaccine Ingelvac CircoFLEX® (vaccine) or aqueous polymer adjuvant cell culture supernatant (placebo) by intramuscular injection into the neck around weaning (2 to 3 weeks of age). Weaning and transfer to the nurseries were performed the day after vaccination (2 to 3 weeks of age); pigs were transferred to the fattening units at 9 weeks of age. All animals (vaccinated or not) were kept under conventional housing conditions and were mixed in pens to ensure that all study pigs were housed in similar conditions, received the same feed and were subjected to the same management practices. At each location change, animals were newly mixed and randomly assigned to the pens according to the usual farm procedure.

### Sample collection and study parameters

Blood samples were collected on the day of inclusion from all piglets, coinciding with the moment of weaning (2 to 3 weeks of age), and prior to injection (vaccine or placebo) to determine the presence of PCV-2 antibodies acquired from maternal colostrum (PCV-2 titre). All animals were also individually weighed at inclusion and before slaughter (about 3 and 25 weeks of age). Only data from live/ear-tagged animals at the end of the study were used to carry out further analysis (5563 animals [91%]; 2835 and 2728 from vaccinated and control groups, respectively).

For quantification of PCV-2 viremia, blood samples from 15% of randomly pre-selected study animals, chosen as representative sample animals (total of 956 piglets; 484 from the vaccinated group and 472 from the control group), were collected on weekly or bi-weekly basis throughout the study period.

### PCV-2 maternally derived antibody (MDA) titre

Quantification of the titre of anti-PCV-2 antibodies in porcine serum samples from the first blood sampling was performed at bioScreen GmbH (Münster, Germany), using an indirect fluorescence antibody titration (IFAT) assay. Briefly, 2–6 × 10^4^ PCV-2 susceptible cells (VIDO-R1 cells [53, 54]) were seeded onto a 96-well plate at 2–6 × 10^4^ cells/well, and inoculated with PCV-2 virus (10^4.5^ TCID_50_/well) for approximately 48 h. After fixation of the cells with ethanol, serial dilutions of porcine serum samples were added to the plates in triplicate and incubated for 1 h at 37 °C, allowing antibodies to bind if present in the sera. Plates were washed and stained for 1 h at 37 °C with a goat-anti-swine FITC-labelled antibody (Dianova, Germany, #114–095-003), which allowed antigen detection in infected cells using fluorescence microscopy. The plates were read by an independent blinded investigator and individual wells reported as positive or negative. Serum antibody titres were calculated by the method of Reed and Muench using the highest dilution still showing specific IFAT reactivity and the number of positive wells per dilution. The method allowed the detection of antibody titres in a range from 1:5 to 1:20480. For the analysis of MDA titres against PCV-2, the data were transformed to base 10 logarithm (log_10_) [25]. As indicated above a total of 5563 animals were used (2835 and 2728 from vaccinated and control groups, respectively).

According to the MDA titre results (those from the first sampling at 2 to 3 weeks of age), the animals were classified into two different groups: “high” (≥2.5 log_10_) and “low” (< 2.5 log_10_) at the time of vaccination. In addition, a third group was established including the 10% of the piglets with the highest antibody titres, whose limit was established by the 90th percentile (≥3.7 log_10_).

### Average daily weight gain (ADWG)

Weight gain was established as a primary parameter of efficacy. Average daily weight gain (kg/day) of each animal was calculated as the difference between the body weights of two weighing time points divided by the number of days between these two weighing time points. For each of the four trials analysed, the mean of ADWG was performed on the basis of the ADWG of only the surviving animals per treatment group. In the same way the mean ADWG was calculated using all the data as a single set. As indicated above, a total of 5563 animals were used (2835 and 2728 from vaccinated and control groups, respectively).

### PCV-2 viral load in serum

Quantification of the PCV-2 viral load in serum was performed at bioScreen GmbH, (Münster, Germany). Samples were analysed in triplicate by a real-time polymerase chain reaction method as described previously [55]. PCV-2 DNA quantification was achieved by comparison of the unknown sample with a standard curve derived from known amounts of PCV-2 ORF plasmid DNA (10^4^–10^12^ copies/ml, 10- fold dilution steps) and the cut-off level for a positive sample was set as 10^4^ template copies per millilitre serum based on validation experiments.

### Analysis of PCV-2 viremia parameters

The mean viral load for each piglet was calculated as the arithmetic mean of individual viral loads (genomic equivalents (gE)/ml of serum) of each sampling day. Only animals that could consistently be followed over the course of the study were included in this calculation (surviving pigs at the end of the study samples from 15% of randomly pre-selected study animals (total of 956 piglets; 484 from the vaccinated group and 472 from the control group).

For a better understanding of the viraemia dynamics in the present study, PCV-2 qPCR data were also analysed using the area under the curve (AUC), as an indicator of the viral load over the time of this study. AUC was calculated using the time of sampling and the PCV-2 viral load quantified using the trapezoidal method, as previously described [47]. In order to analyse differences between study groups, the mean value of AUC was calculated for each of the populations.

### Mortality

Dead animals during the study in each of the farms were registered by the staff of the same. For each animal, their identification number was recorded as well as the date of death or withdrawal from the study.

### Statistical analyses

All statistical analyses were carried out using the SAS system V.9.1.3 (SAS institute Inc., Cary, NC, USA). For all analyses, the individual pig was considered as the experimental unit. The farm was included as a variable in the analysis. The significance level (α) was set at 0.05.

#### Evaluation of the efficacy of the vaccination protocol

A basal homogeneity analysis was performed to verify that the treatment groups were balanced by sex and weight prior the beginning of the trial. Parameters ADWG, AUC and mortality were analysed for assumption of normality and homogeneity of variances using the Kolmogorov-Smirnov and Levene test, respectively. Differences between groups for ADWG, AUC and mortality were compared using an ANOVA test for normally distributed variables and Kruskall-Wallis test for non-normally distributed ones.

#### Maternally derived antibody effects on ADWG

A linear regression was performed to evaluate the strength of association between the PCV-2 antibody titre at the day of vaccination for vaccinated and control piglets and the ADWG during the whole rearing period (from 4 to 25 weeks of age). Moreover, a similar linear regression analysis was performed for vaccinated piglets but splitting the animals in low (< 2.5 log_10_), high (≥2.5 log_10_) and extremely high (≥3.7 log_10_) PCV-2 titres, as described above.

#### Maternally derived antibody effects on viremia (PCV-2 AUC)

Differences between groups (placebo-treatment and vaccinated animals) for AUC were performed splitting the animals in low (< 2.5 log_10_), high (≥2.5 log_10_) and extremely high (≥3.7 log_10_) PCV-2 titres, as described above.

#### PCV-2 AUC effects on ADWG

A linear regression was performed to evaluate the strength of association between the PCV-2 AUC for vaccinated and control piglets and the ADWG during the whole rearing period (from 4 to 25 weeks of age).

## Data Availability

Not applicable.

## Abbreviations

ADWG: Average daily weight gain
AUC: Area under the curve
IAV: Influenza A virus
IFAT: Indirect fluorescence antibody test
IPMA: Immunoperoxidase monolayer assay
MDA: Maternally derived antibody
MDI: Maternally transferred immunity
PCV-2: Porcine circovirus 2
PCV-2-ED: Porcine circovirus 2 enteric disease
PCV-2-LD: Porcine circovirus 2 lung disease
PCV-2-RD: Porcine circovirus 2 reproductive disease
PCV-2-SD: Porcine circovirus 2 systemic disease
PCV-2-SI: Porcine circovirus 2 subclinical infection
PCVDs: Porcine circovirus diseases
PDNS: Porcine dermatitis and nephropathy syndrome
PMWS: Post-weaning multisystemic wasting syndrome
SEM: Standard error of the mean
